# Comparative Analysis of Hepatic CD14 Expression between Two Different Endotoxin Shock Model Mice: Relation between Hepatic Injury and CD14 Expression

**DOI:** 10.1371/journal.pone.0053692

**Published:** 2013-01-07

**Authors:** Hiroyasu Hozumi, Rui Tada, Taisuke Murakami, Yoshiyuki Adachi, Naohito Ohno

**Affiliations:** Laboratory for Immunopharmacology of Microbial Products, School of Pharmacy, Tokyo University of Pharmacy and Life Sciences, Hachioji, Tokyo, Japan; Harvard Medical School, United States of America

## Abstract

CD14 is a glycoprotein that recognizes gram-negative bacterial lipopolysaccharide (LPS) and exists in both membrane-bound and soluble forms. Infectious and/or inflammatory diseases induce CD14 expression, which may be involved in the pathology of endotoxin shock. We previously found that the expression of CD14 protein differs among the endotoxin shock models used, although the reasons for these differences are unclear. We hypothesized that the differences in CD14 expression might be due to liver injury, because the hepatic tissue produces CD14 protein. We investigated CD14 expression in the plasma and liver in the carrageenan (CAR)-primed and D-gal*N*-primed mouse models of endotoxin shock. Our results showed that severe liver injury was not induced in CAR-primed endotoxin shock model mice. In this CAR-primed model, the higher mRNA and protein expression of CD14 was observed in the liver, especially in the interlobular bile duct in contrast to D-gal*N*-primed-endotoxin shock model mice. Our findings indicated that the molecular mechanism(s) underlying septic shock in CAR-primed and D-gal*N*-primed endotoxin shock models are quite different. Because CD14 expression is correlated with clinical observations, the CAR-primed endotoxin shock model might be useful for studying the functions of CD14 during septic shock *in vivo*.

## Introduction

Despite advances in medicine that have resulted in the successful control of infections caused by gram-negative bacteria, mortality associated with septic shock (sepsis) still ranges from 30% to 50% [1], [2]. In the United States alone, there are 210,000 cases of sepsis every year [3]. Two major reasons have been identified for the high mortality rate of septic shock. First, while several new treatments are under investigation, none are readily available in clinical settings [4], [5]. Second, there are no methods for early diagnosis of septic shock. Prompt diagnosis of septic shock in the early stage is quite important for effective treatment of sepsis [6].

Lipopolysaccharide (LPS), which is a structural component of gram-negative bacteria, plays a central role in septic shock [7]–[9]. LPS consists of lipid A, which is widely conserved in gram-negative bacteria, a core region, and an O-antigen region. In experimental animal models of endotoxin shock, administration of purified LPS induces numerous pathophysiological changes that can lead to severe shock called “sepsis” and death [10]. Most of these changes, including the release of inflammatory cytokines, are induced by the recognition of the lipid A region by the toll-like receptor 4 (TLR4) [11]–[13].

In addition, during the course of LPS recognition, CD14 molecule is known to play a crucial role for this process. CD14 is expressed on the surfaces of monocytes and neutrophils as a 55-kDa glycosylphosphatidylinositol (GPI)-anchored membrane protein (mCD14), and is also present in the serum as a soluble isoform (sCD14) [14]. Although there is only one mRNA transcript of the CD14 gene, two different types of sCD14 have been characterized based on its molecular weight. First, a 48–49 kDa of sCD14 which may be shedded by the activation of a membrane-associated serine protease was identified. Second, a 55–56 kDa of sCD14, escaping GPI anchor attachment with the C-terminal peptide was also found [15]. Both mCD14 and sCD14 play central roles in LPS recognition. CD14 can recognize the complex of LPS-binding protein (LBP) and LPS, and then transfer this complex to the TLR4/MD-2 complex. MD-2 is also known as lymphocyte antigen 96 (LY96) and ESOP-1. Subsequent signals through TLR4/MD-2 activate host immune cells such as monocytes and macrophages, thereby inducing the inflammatory mediators, namely, tumor necrosis factor-alpha (TNF-α), interleukin-1β (IL-1β), and IL-6 [16]–[18]. Moreover, sCD14, producing by hepatocytes as well as monocytes, would act as a receptor for the LPS against the cells, which does not express the surface CD14 molecule such as endothelial and epithelial cells [15], [19]. These inflammatory cytokines are critical for the pathogenicity of septic shock, indicating that CD14 has a crucial role in the progression of septic shock caused by gram-negative bacteria. In septic shock, the sCD14 level is dramatically increased, leading us to hypothesize that the sCD14 level might be a marker of septic shock during its early stage, and thus, a potential diagnostic tool [20].

We previously reported that plasma sCD14 levels differed between carrageenan (CAR)-primed- and D-galactosamine (D-gal*N*)-primed endotoxin shock model mice. In CAR-primed endotoxin shock model mice, sCD14 levels are significantly increased over those in the D-gal*N*-primed model, implying that the mechanism(s) underlying these 2 models are different, even though both methods are used to model endotoxin shock [21]. Severe liver injury and high sensitivity to TNF-α were observed in the D-gal*N*-primed endotoxin shock-induced mice, and these factors may contribute to the high mortality observed in this model. On the other hand, the CAR-primed endotoxin shock-induced model is not well understood [22]. Expression of sCD14 in the livers of both CAR-primed and D-gal*N*-primed endotoxin shock model mice has not been reported despite the fact that the hepatic tissue produces CD14 protein [23]. These facts led us to investigate the relationship between liver injury and CD14 expression in these two endotoxin shock-induced models. During endotoxin shock, the plasma sCD14 levels increase, and the animal shows typical symptoms such as multiple organ failure (MOF), including liver injury and systemic inflammatory response syndrome (SIRS). Therefore, this analysis may provide useful tools for early prediction of endotoxin shock [24], [25]. Moreover, CAR-primed endotoxin shock model allows us to gain insight into the role of sCD14 and its relationship with changes in CD14 levels and progression of endotoxin shock symptoms. In the present study, we demonstrated the increased CD14 expression in the hepatic tissue, in CAR-primed endotoxin shock model mice.

## Results

### Lethality and the production of IL-6 in plasma of endotoxin shock model system in carrageenan-primed C57BL/6 mice

**Figure 1:**
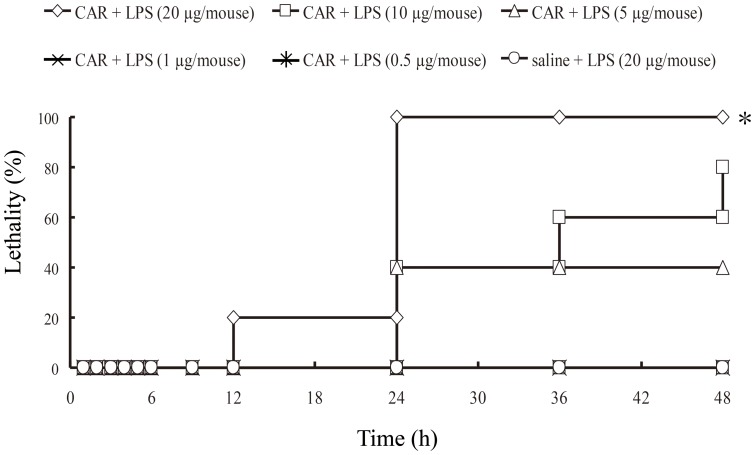
Lethality of CAR-primed endotoxin shock model mice at various doses of LPS. Various doses of LPS were administered i.v. to CAR-primed or unprimed C57BL/6 mice. CAR (5 mg/mouse) was administered i.p. 24 h prior to LPS injection. Survival was observed for 2 days (n = 5). Significant difference from non-primed group: *p<0.05.

**Figure 2:**
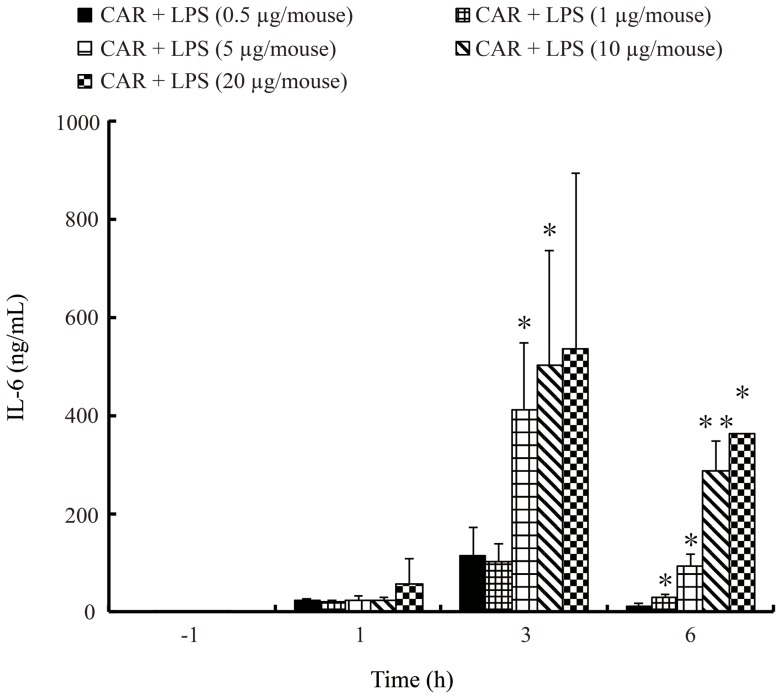
Cytokine production in the plasma of CAR-primed endotoxin shock model mice at various doses of LPS. Various doses of LPS (0.5, 1, 5, 10, or 20 μg/mouse) were administered i.v. to CAR-primed or unprimed C57BL/6 mice. CAR (5 mg/mouse) was administered i.p. 24 h prior to LPS injection. Thereafter, plasma was collected at 0, 1, 3, and 6 h, and the IL-6 concentrations was determined using an ELISA system. The data obtained were representative of at least 3 independent experiments and are expressed as mean ± standard deviation (SD) values for samples assayed in triplicate. Significant difference from 0.5 μg/mouse of LPS administrated mice: *p<0.05; **p<0.01.

We first examined the dose of LPS required for lethal toxicity in CAR-primed C57BL/6 mice. In the CAR-primed mice, LPS injection induced endotoxin shock in a dose-dependent manner (Figure 1). Next, we examined the plasma cytokine levels, since it was reported that inflammatory cytokines such as IL-6 are significantly increased during endotoxin shock. Our results show that plasma IL-6 levels in CAR-primed mice were significantly increased at 3 h after LPS-injection in dose-dependent manner (Figure 2).

### sCD14 level in the plasma of CAR-primed endotoxin shock model mice

**Figure 3:**
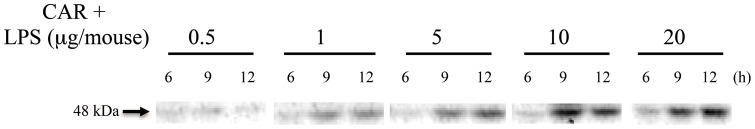
sCD14 level in the plasma of CAR-primed endotoxin shock-induced model mice at various doses of LPS. Plasma was collected from CAR-primed C57BL/6 mice that were administered with LPS at the indicated time. Soluble CD14 in the plasma was detected as follows: Each plasma sample was diluted 20 folds with saline, and then subjected to SDS-PAGE and subsequent western blotting. Then, sCD14 on the membrane was detected using anti-mouse CD14 polyclonal antibody.

In our previous study, we investigated the level of sCD14 in the plasma of CAR-primed endotoxin shock model using the closed colony ICR mice. In this model, plasma sCD14 levels dramatically increased during endotoxin shock [21]. Although hepatic cells reportedly produce sCD14 [23], CD14 expression in animal models of endotoxin shock was unclear. Therefore, we explored CD14 expression in C57BL/6 mice. We examined plasma sCD14 levels using western blotting analysis. In CAR-primed mice, the plasma sCD14 level increased within 6 h of LPS injection, and reached the peak at 12 h after injection in a dose-dependent manner (Figure 3). sCD14 levels matched the production of proinflammatory cytokine. Therefore, sCD14 levels in plasma might be involved in the pathology and/or used as an early diagnostic tool for this model.

### Comparison of liver injury in endotoxin shock models

**Figure 4:**
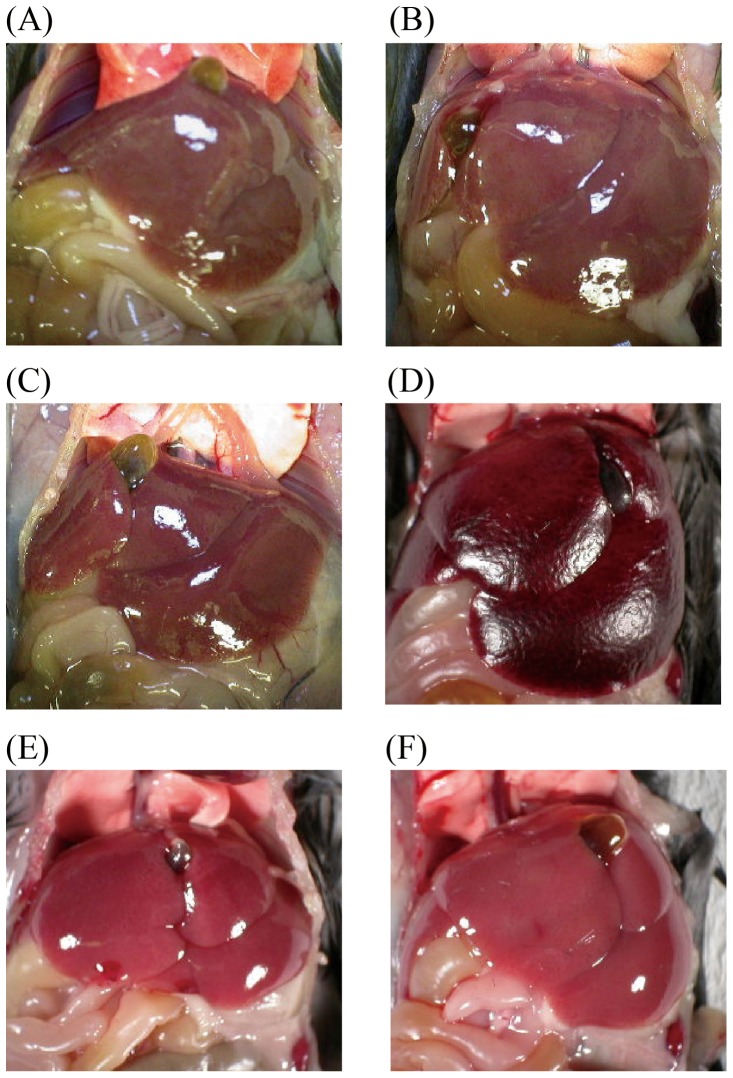
Observation of hepatic injury in CAR- and D-gal*N*-primed endotoxin shock-induced mice. All mice were abataged at indicated times, and photographed. (A) After 12 h of injecting LPS (20 μg/mouse) to the CAR (5 mg/mouse)-primed mice liver (B) After 36 h of injecting LPS (20 μg/mouse) to CAR (5 mg/mouse)-primed mice liver (C) After 12 h of injecting LPS (20 μg/mouse) to non-primed mice liver (D) After 6 h of injecting LPS (0.5 μg/mouse) to D-gal*N* (20 mg/mouse)-primed mice liver (E) After 6 h of injecting only D-gal*N* (20 mg/mouse) (F) Control mice liver.

It was reported that the D-gal*N*-primed model of endotoxin shock showed severe liver injury. This injury is considered as to be a major reason for the lethality observed in this model [26], [27]. In contrast, there was no such a report in cases of CAR-primed endotoxin shock despite the fact that liver injury may be important in the pathogenesis of sepsis. Therefore, we examined liver injury in these 2 models. Microscopic analyses indicated that liver injury in CAR-primed mice was less severe than that observed in D-gal*N*-primed mice (Figure 4). These data imply that endotoxin shock occurs through different mechanisms in these models.

### Histological examination of the livers from endotoxin shock model mice

**Figure 5:**
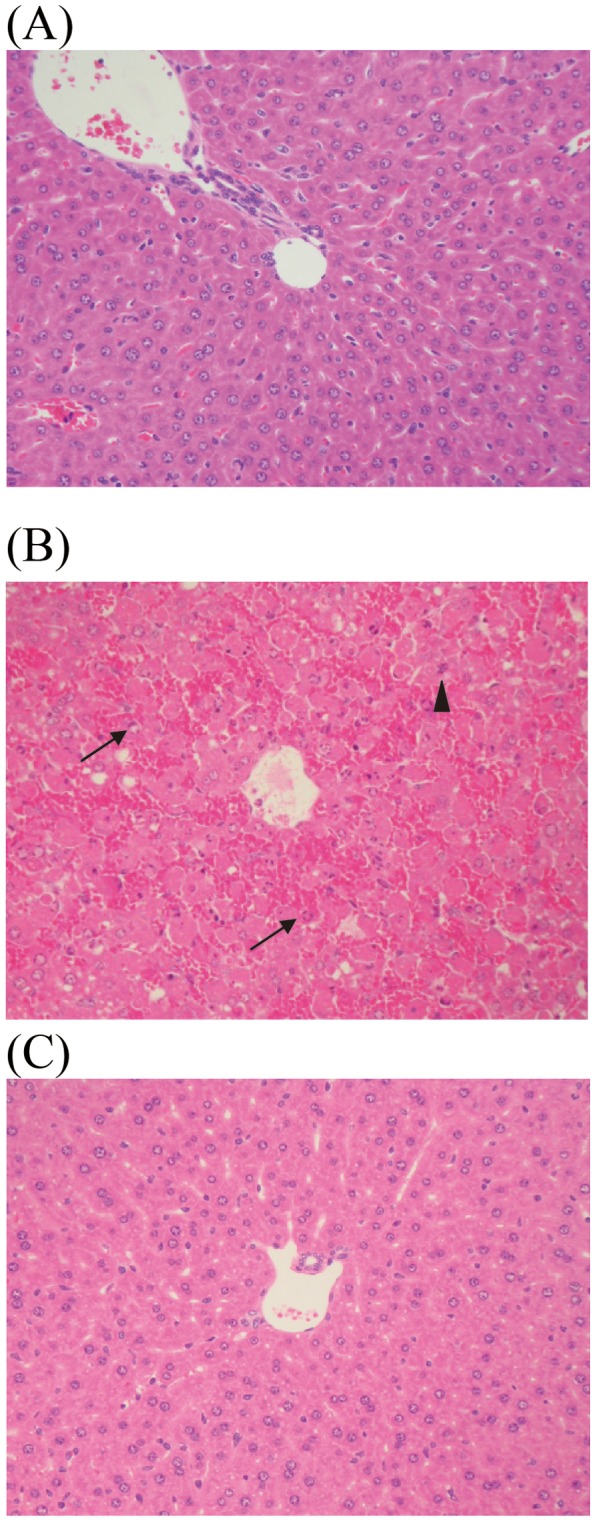
Histological examination of CAR- or d-gal*N*-primed endotoxin shock-induced mice liver. (A) LPS (20 μg/mouse) was administered i.v. to CAR (5 mg/mouse)-primed C57BL/6 mice, and 12 h later, the livers were extirpated from these mice. (B) LPS (0.5 μg/mouse) was administered i.v. to D-gal*N* (20 mg/mouse)-primed C57BL/6 mice. The livers were extirpated after 6 h of LPS injection. (C) Untreated mice were used as negative control. The extirpated livers were fixed in 10%-formaldehyde neutral buffer solution and embedded in paraffin; thin sections were cut and stained with hematoxylin-eosin. Arrow and arrow head represent chromatin aggregation and nuclear fragmentation, respectively. Magnification: ×200.

**Figure 6:**
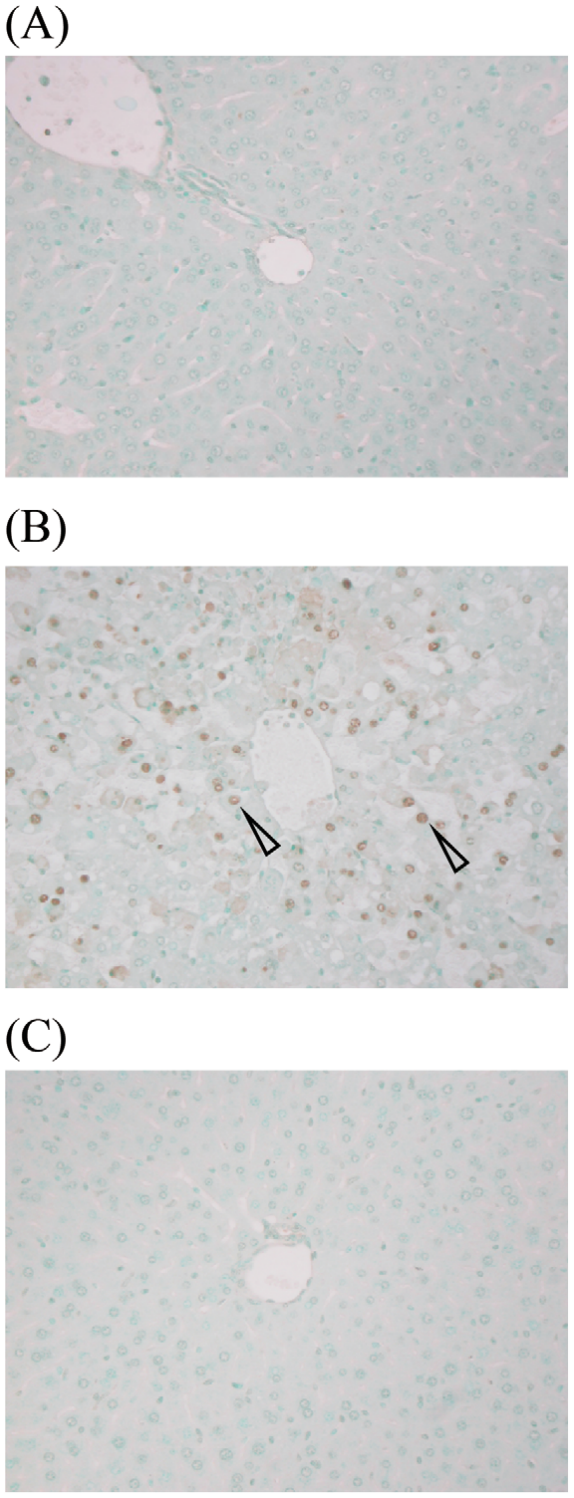
TUNEL staining of CAR- or D-gal*N*-primed endotoxin shock-induced mice liver. (A) LPS (20 μg/mouse) was administered i.v. to CAR (5 mg/mouse)-primed C57BL/6 mice, and their livers were extirpated after 12 h of LPS injection. (B) LPS (0.5 μg/mouse) was administered i.v. to D-gal*N* (20 mg/mouse)-primed C57BL/6 mice, and their livers were extirpated after 6 h of LPS injection. (C) Untreated mice were used as negative control. Arrow represents apoptotic cells. Magnification: ×200.

**Figure 7:**
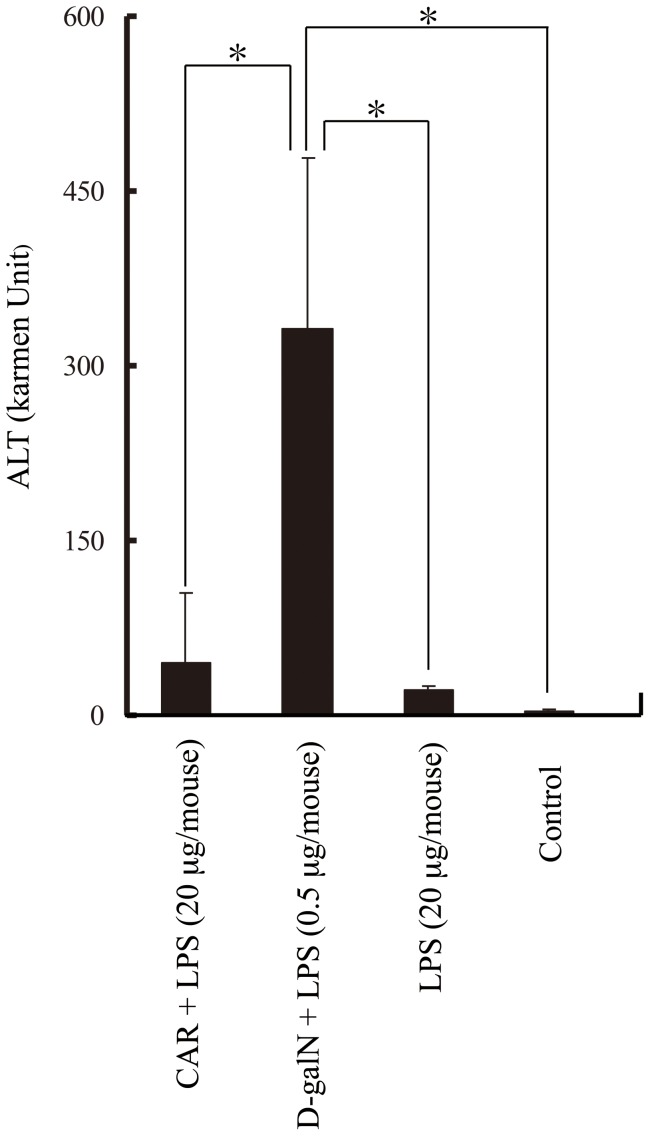
ALT level in the sera of the CAR- or d-gal*N*-primed endotoxin shock-induced mice. The sera samples were collected from CAR-primed C57BL/6 mice administered with LPS at 12 h or D-gal*N*-primed C57BL/6 mice administered with LPS at 6 h. ALT level was determined by Transaminase C2 test kit, as described in Materials and methods section. The data obtained were representative of at least 3 independent experiments and are expressed as mean ± SD values for samples assayed in triplicate. Significant difference: *p<0.05.

Next, we examined liver injuries using hematoxylin-eosin (HE) staining and terminal deoxynucleotidyl transferase-mediated dUTP nick end labeling (TUNEL) staining. No histological differences were noted between HE-stained CAR-primed mice and control mice (Figure 5). However, in the liver from D-gal*N*-primed endotoxin shock model mice, we observed fragmented hepatocytes and chromatin aggregation. Nuclear fragmentation was also observed in the nuclei, strongly indicating that the liver cells of D-gal*N*-primed mice, but not CAR-primed mice, were apoptotic. Therefore, we examined whether apoptotic cells can be detected by TUNEL staining (Figure 6). We did not detect apoptotic cells in the livers of the CAR-primed endotoxin shock model mice or control mice (Figure 6A and C). However, as expected, we observed apoptotic cells in livers of D-gal*N*-primed mice (Figure 6B). We also confirmed liver injuries based on the plasma ALT levels. We showed that plasma ALT levels were markedly increased in the D-gal*N*-primed, whereas those in the CAR-primed mice were much lower (Figure 7). These observations confirmed the results of histological examinations using HE staining and TUNEL staining of liver tissue.

### Immunohistochemical staining for sCD14 in the livers of endotoxin shock model mice

**Figure 8:**
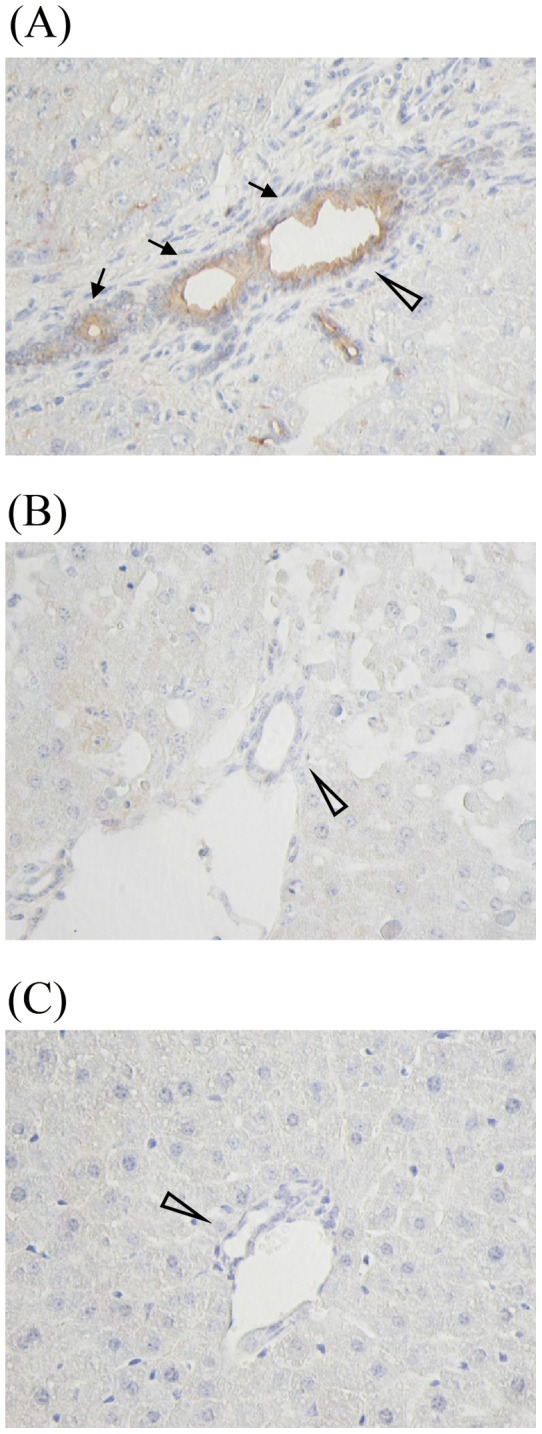
Immunohistochemical staining of CD14 in CAR- or D-gal*N*-primed endotoxin shock-induced mice liver. Liver specimens were collected from CAR-primed C57BL/6 mice after LPS administration at 12 h or D-gal*N*-primed C57BL/6 mice after LPS administration at 6 h. These hepatic tissues were immunohistochemically studied by staining with the biotinylated anti-mouse CD14 monoclonal antibody 4C1. (A) Following priming with CAR (5 mg/mouse), these mice were injected with LPS (20 μg/mouse). (B) The mice were simultaneously injected with D-gal*N* (20 mg/mouse) and LPS (0.5 μg/mouse). (C) The mice were injected with saline. CD14 expression was observed (arrow) in the area surrounding the bile duct (white arrowhead). Magnification: ×200.

The relationship between hepatic injury and sCD14 expression in the liver tissue in endotoxin shock model is still unclear. We next examined the expression of CD14 in the mouse liver immediately after death caused by endotoxin shock. As shown in Figure 8, liver sections of the CAR-primed endotoxin shock model after LPS injection showed the expression of CD14 at interlobular bile ducts (Figure 8A). In contrast to the CAR-primed model mice, D-gal*N*-primed (Figure 8B) and LPS-treated (Figure 8C) mice did not express CD14.

### Expression of sCD14 in bile in endotoxin shock model mice

**Figure 9:**
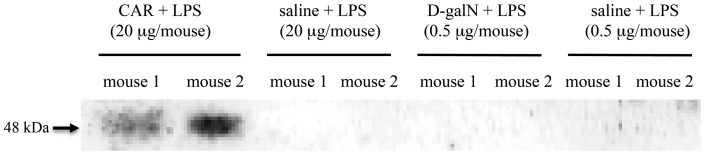
sCD14 expression in bile. Bile samples were collected from the gallbladder 12 h after CAR-primed C57BL/6 mice were injected with LPS (A), after D-gal*N*-primed C57BL/6 mice were injected with LPS (B), and from the negative control (C). Each sample was diluted with saline, and then subjected to SDS-PAGE and subsequent western blot analysis. The expression of sCD14 on the nitro cellulose membrane was detected using an anti-mouse CD14 polyclonal antibody as a probe.

**Figure 10:**
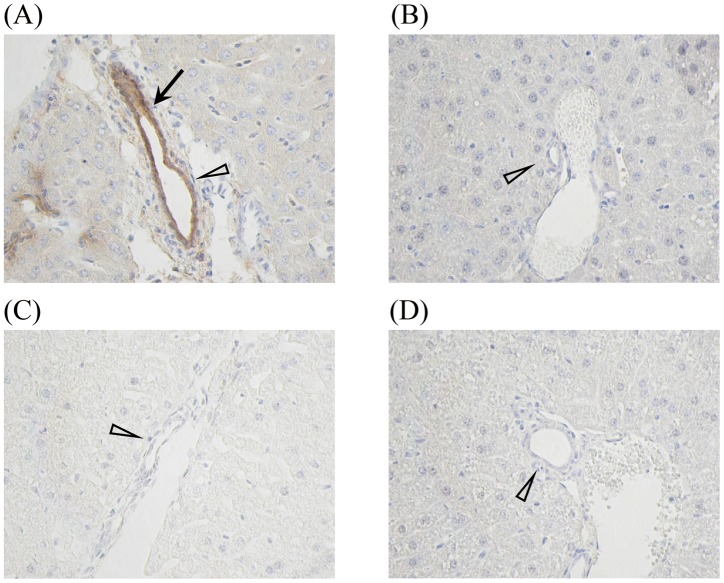
Immunohistochemical staining of CAR- and D-gal*N*-primed and control mice liver. The immunohistochemistry of the mice hepatic tissues was studied by staining with the biotinylated anti-mouse CD14 monoclonal antibody (10 μg/mL) 4C1. The tissues were collected from CAR-primed C57BL/6 mice that were injected with LPS at 12 h or from the control mice and the transfected plasma was collected from the CAR-primed mice that were injected with LPS at 12 h or from the control mice. C57BL/6 mice were injected with LPS at 6 h. (A) The mice were primed with CAR (5 mg/mouse) and injected with LPS (20 μg/mouse). (B) The liver of the mice that intravenously received the plasma prepared from CAR-primed and LPS-injected mice. (C) The liver of an untreated mouse was used as control. (D) The livers were extirpated from the mice that received control plasma, prepared from control mice. CD14 expression (arrow) was observed in the area surrounding the bile duct (white arrowhead). Magnification: ×200.

**Figure 11:**
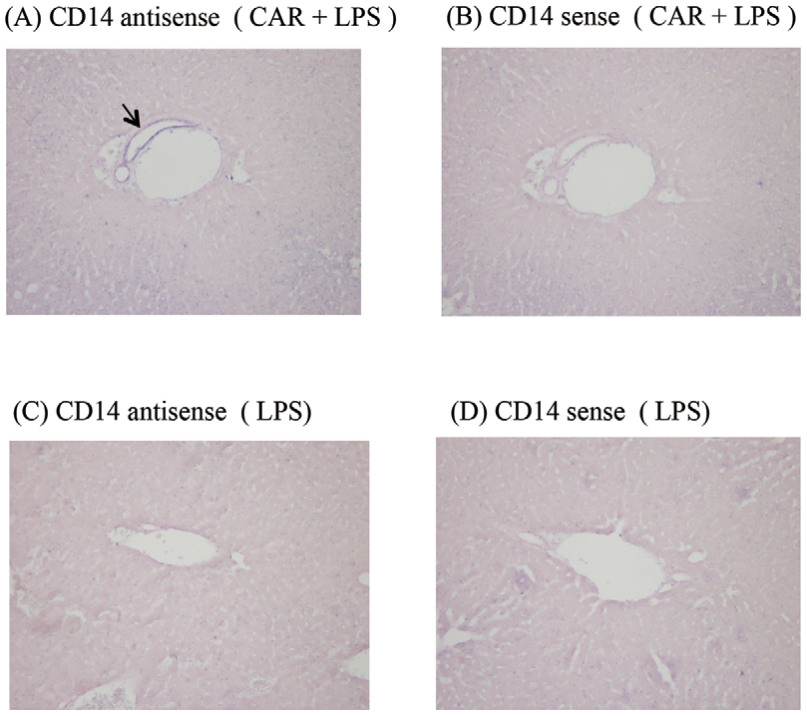
Gene expression of CD14 in the mice liver. The gene expression of CD14 in the mice liver was determined by *in situ* hybridization with CD14 sense and antisense probes. (A) Liver specimens from CAR-primed endotoxin shock-induced mice stained using CD14 antisense probe (B) Liver specimens from CAR-primed endotoxin shock-induced mice stained using CD14 sense probe (C) Liver specimens from mice injected with LPS (20 ng/mouse) stained using CD14 antisense probe (D) Liver specimens from mice injected with LPS (20 ng/mouse) stained using CD14 sense probe. Arrow represents CD14 mRNA expression. Magnification: ×200.

To confirm sCD14 expression in bile, we assessed whether the sCD14 protein within the gallbladder could be detected. Bile samples collected from the gallbladders of CAR-primed LPS-injected mice showed positive bands on the membrane after western blotting (Figure 9). The CD14 expression observed at the interlobular ducts might be due to the deposits around the interlobular bile duct. Therefore, we examined CD14 expression in the biliary region. The bile ducts of CAR-primed mice stained positive for CD14 (Figure 10A), but the bile ducts of the mice that received plasma from the CAR-primed mice did not (Figure 10B). These results suggested that the presence of CD14 was not due to the deposition of circulating sCD14 in the epithelial cells of the bile duct. In addition, we examined the gene expression of CD14 at the interlobular bile duct of the liver by using *in situ* hybridization. As shown in Figure 11, CD14 expression at the interlobular bile ducts of the CAR-primed mice was detected by a CD14 antisense probe (Figure 11A), but this expression was not detected in the control mice that received only LPS (Figure 11B). Taken together, these results strongly indicate that CD14 is expressed directly around the interlobular bile ducts as well as in bile.

## Discussion

In this study, we demonstrated the following: (1) In CAR-primed endotoxin shock model mouse, although the liver injury was not severe, it dramatically induced CD14 expression in both the liver and plasma; (2) In contrast to the CAR-primed endotoxin shock model mice, intense liver injury, including cell apoptosis, was observed in the D-gal*N*-primed model, and it did not induce CD14 expression; and (3) The expression of CD14 protein and mRNA in the livers of the CAR-primed endotoxin shock model mice was detected especially in the interlobular bile ducts. On the basis of these findings, we elucidate that the molecular mechanism(s) underlying septic shock in CAR-primed and D-gal*N*-primed endotoxin shock model mice are entirely different. From a clinical point of view, the CAR-primed endotoxin shock model might be more suitable for investigating the functions of CD14 during septic shock *in vivo* and for early diagnosis of septic shock using CD14.

Many studies have revealed the role of CD14 in recognizing LPS, and in complex formation with TLR4/MD-2 [11], [17]. In addition, we found that *in vivo* CD14 expression during shock symptoms differed between the endotoxin shock models [21]. However, the reasons underlying these differences are not well understood. Since the liver tissue was reported to be a candidate CD14-producing organ, we hypothesized that the liver injury caused by endotoxin shock differs between models. Moreover, as for D-gal*N*-primed endotoxin shock model, detailed studies conducted until date have the following findings: (1) This model exhibits severe liver injury due to induction of apoptosis, (2) it enhances the responsiveness to inflammatory cytokines such as TNF-α, and (3) it does not induce CD14 expression in the plasma during shock [21], [26]–[28]. On the other hand, considering the CAR-primed endotoxin shock model, few studies have shown that (1) increased inflammatory cytokines production might be related to the enhancement of its lethality rather than responsiveness to TNF-α and (2) CD14 expression in plasma during shock was dramatically increased [21], [22], [29]. Our hypothesis also seems to support these facts.

Here, we demonstrated that the CAR-primed endotoxin shock model mice show increased production of the inflammatory cytokine IL-6, and also increased expression of CD14 protein in the plasma (Figures 1, 2, and 3). Importantly, as for the serum levels of sCD14, Aguiar *et* *al*. reported that patients with increased sCD14 in plasma show an early onset of septic symptoms [25]. In addition, the level of sCD14-subtype, which is produced in association with infection and specifically expressed in sepsis, is much higher in these subjects than in the serum from healthy volunteers [30]. These facts suggest that the plasma levels of sCD14 in the CAR-primed endotoxin shock model mice mirror those in humans. sCD14 may thus play a pivotal role for the septic pathophysiology. With respect to liver injury, which is reported to be the organ producing CD14, no injury was observed in the CAR-primed shock model mice (Figures 4, 5, 6, and 7). The CAR-primed model showed high level of CD14 expression with slight liver injury, whereas the D-gal*N*-primed model showed low level of CD14 expression with a severe liver injury. We hypothesized that the liver might be responsible for the production of CD14. As expected, CD14 would be expressed directly around the interlobular bile ducts and in bile (Figures 8, 9, 10, and 11). CD14 expression in the liver tissue was reported in biliary atresia (BA) patients and in rats that received surgery for bile duct ligation in order to generate a BA animal model. The BA patients showed increased levels of CD14 and endotoxin in the plasma. The BA rat model showed the expression of CD14 protein and mRNA in the parenchyma of the liver tissue after ligation of the bile duct [31]. However, in the present study, the biliary duct epithelial cells rather than the liver parenchyma cells or the Kupffer cells showed strong positive signals in immunostaining or in the *in situ* study in the CAR-primed endotoxin shock model. In the liver, besides the hepatocytes, nonparenchymal cells such as the Kupffer cells, endothelial cells, and neutrophils can also express CD14 mRNA and subsequent synthesize CD14 protein [23], [32], [33]. In addition to those cells, the biliary duct epithelial cells also express CD14 protein and mRNA in the liver tissues of the CAR-primed mouse (Figure 9, 10, and 11). These data suggest that the biliary epithelial cells are sources of the CD14 observed in the bile (Figure 10). Soluble CD14 can mediate LPS signal transduction in the endothelial cells that lack the membrane form of the receptor [34]. Expression of TLR4 and related molecules capable of recognizing LPS in the biliary epithelial cells is demonstrated by immunostaining and RT-PCR [35]. The sCD14 molecules in the biliary circulation and plasma may influence LPS susceptibility of the epithelial cells and systemic innate immune cells in CAR-primed mice.

Although the proposed role of CAR in LPS-induced sCD14 expression needs more evidence, systemic administration of CAR may block the reticuloendothelial system (RES). CAR is preferentially incorporated by the Kupffer cells in the liver [36]. Administration of CAR to animals also creates an experimental model of jaundice [37]. RES dysfunction resulting from biliary obstruction might be an important cofactor in the pathogenesis of bile-induced pancreatitis [38]. Thus LPS administration may induce multiple organ failure, including pancreatitis [39]. Treatment with CAR may induce dysfunction of phagocytes and biliary ducts responsible for elimination of the LPS in circulation, and may increase LPS sensitivity.

The present study showed that CD14 produced by the liver dramatically increased in the CAR-primed endotoxin shock model. The etiology of sepsis is definitely multi-factorial; however, findings of the present study suggest that the CAR-primed endotoxin shock model could be useful for shedding light on the *in vivo* function of CD14, because this model shows strong CD14 expression in both the plasma and liver tissue with no apoptosis in the liver.

## Materials and Methods

### Ethics Statement

All animal experiments followed the guidelines for laboratory animal experiments provided by the Tokyo University of Pharmacy and Life Sciences (TUPLS), and each experimental protocol was approved by the Committee for Laboratory Animal Experiments at TUPLS.

### Animals

Female C57BL/6 mice (6 weeks old) were purchased from Japan SLC, Inc., (Shizuoka, Japan). The mice were housed in a specific pathogen-free environment.

### Chemicals

Phenol-extracted *Escherichia coli* (O111: B4)-derived LPS and iota-carrageenan (CAR) (Sigma-Aldrich Co., St. Louis, MO) were dissolved in pyrogen-free physiological saline (Otsuka Pharmaceutical Co., Naruto, Japan). The CAR solution was autoclaved at 121°C for 15 min before use. D-Galactosamine hydrochloride (Wako Co.,) was dissolved in pyrogen-free physiological saline and sterilized using 0.2-μm syringe filter before use.

### Endotoxin shock model mice

CAR-primed endotoxin shock model mice were prepared as previously reported [19]. Briefly, CAR (5 mg/mouse) was administered i.p. to female C57BL/6 mice, and then, after 24 h, LPS (0.5, 1, 5, 10, and 20 μg/mouse) was administered. In case of D-gal*N*-primed endotoxin shock model mice, D-galactosamine (20 mg/mouse) was administered i.p. to female C57BL/6 mice, immediately followed by LPS (0.5 μg/mouse) administration as reported previously [40]. These values and the subsequent mortality were recorded until 48 h after LPS injection. The data showed was one representative of at least 3 independent experiments.

### Cytokine assay

Cytokine concentrations in the plasma were determined using an OptiEIA kit (BD Biosciences). The data obtained were representative of at least 3 independent experiments and are expressed as mean ± standard deviation values for samples assayed in triplicate.

### Preparation of sCD14

The plasmid expression vector containing the cDNA coding for amino acids 1–320 of mouse CD14 was transfected into EBNA293 cells. Recombinant mouse CD14 in the culture supernatant was purified by Ni-nitrilotriacetic acid agarose (Qiagen, Basel, Switzerland) chromatography using elution buffer containing imidazole (250 mM).

### Preparation of anti-mouse CD14 polyclonal antibody

The CD14 fragment was obtained from the lysate of *E. coli* BL21, which was transformed with a plasmid containing the cDNA coding for amino acids 161–320 of mouse CD14. The purified CD14 fragment was injected s.c. into a rabbit. After that, anti-mouse CD14 antibody was purified from the rabbit serum by using protein A-conjugated agarose. The antibody was biotinylated with Biotin-(AC_5_)_2_-OSu (DOJINDO, Japan).

### Western blotting for the detection of sCD14 in plasma

We have already elucidated the method for detecting sCD14 in plasma and showed a good correlation of its concentration [21]. Plasma samples were collected from endotoxin shock model mice. The samples were mixed with sample buffer containing 10%-2-mercaptoethanol and heated in boiling water for 5 min. The sample thus prepared was analyzed by SDS-PAGE using 10%-gel. Biotin-labeled anti-CD14 rabbit polyclonal antibody diluted in blocking buffer was used for staining the blots, and the bound antibody was detected using anti-biotin HRP-linked antibody (Cell Signaling Technology). The data obtained were representative of at least 3 independent experiments.

### Measurement of alanine aminotransferase (ALT)

Plasma samples collected from endotoxin shock model mice were incubated at room temperature, and then, kept on ice. Then, the samples were centrifuged at 15,000 revolutions per minute (rpm) for 10 min at 4°C, and the supernatant was collected. These samples were assayed for ALT levels (Transaminase CII Test, Wako). The data obtained were representative of at least 3 independent experiments and are expressed as mean ± standard deviation values for samples assayed in triplicate.

### Hematoxylin-eosin (HE) and TUNEL staining

CAR-primed model mice were sacrificed after 12 h of LPS administration, and D-gal*N*-primed endotoxin shock model mice, after 6 h of LPS administration. Their livers were extirpated, fixed in 10%-formaldehyde neutral buffer solution, and decalcified. It was commissioned to SRL, Inc. The livers were embedding in paraffin, cut into sections, and stained with hematoxylin and eosin, and TUNEL. The data showed was one representative of at least 3 independent experiments.

### Immunohistochemical staining for detection of sCD14

CD14 expression in mouse liver was studied by immunohistochemical assays. In brief, the tissue samples were deparaffinized and hydrated, and cut into 6-μm-thick sections on a cryostat microtome (TISSUE-TEK). The sections were dewaxed with xylene and rehydrated with graded ethanol. The slides were washed with PBS, incubated for 30 min at room temperature, and blocked with 10% normal goat serum (DakoCytomation) in PBS. After washing, the samples were treated with biotinylated anti-mouse CD14 monoclonal antibody (10 μg/mL) 4C1 (SEIKAGAKU CORPORATION) for 90 min in room temperature. After washing, the slides were incubated with 0.3% H_2_O_2_-methanol at room temperature for 30 min, and then, with HRP-conjugated streptavidin (1/1000; Pharmingen) at room temperature for 45 min. The visualizing agent used was 0.05 M Tris buffer (pH 7.6) containing diaminobenzidine (DAB, 0.3 mg/mL). The samples were stained with Mayer's hematoxylin solution (Wako) and incubated with methanol (KANTO CHEMICAL CO., INC.,), xylene (Wako). Finally, the samples were enclosed in ENTELLAN neu (Merck, Japan). The slides were rinsed and the specimens were incubated with biotin-labeled goat anti-mouse IgG secondary antibody for 30 min at room temperature, followed by incubation with peroxidase-labeled streptavidin under identical conditions. Then, the DAB substrate was added as the visualizing agent. The data showed was one representative of at least 3 independent experiments.

### Components of plasma

CAR (5 mg/mouse) was administered i.p. to female C57BL/6 mice. Twenty-four hours later, LPS was administered (0.5, 1, 5, 10, and 20 μg/mouse). After 12 h, blood samples were collected using heparin-containing syringes and centrifuged at 15,000 rpm for 10 min at 4°C. Supernatants were inactivated by incubation at 56°C for 30 min. Non-treated mice were injected with 500 μL of plasma sample, which is obtained from CAR-primed endotoxin shock model mice. After 1 h, mice were sacrificed and plasma and liver samples were collected.

### *In situ* hybridization for the gene expression of CD14

*In situ* hybridization was carried out by using paraffin-embedded sections and digoxigenin-labeled cRNA probe (Adbiome Researchtech, Inc., Niigata, Japan). The mouse liver tissues were fixed in 4%-paraformaldehyde in PBS, embedded in paraffin, and cut into serial sections of 8-μm thickness. Digoxigenin-labeled antisense and sense cRNA probes for CD14 were synthesized by *in vitro* transcription using cDNA (500 bp corresponding to the coding region spanning the nucleotide position 232 to 731 bp in the mouse CD14 cDNA sequence). The tissue sections were deparaffinized, re-fixed, treated with acid, and then, subjected to protease digestion with an automatic staining module (Discovery; Roche Diagnostics, Penzberg, Germany). Hybridization was performed with antisense or sense probe (50 ng/mL) at 60°C for 6 h in hybridization solution (Ribohybe, Roche Diagnostics). After washing in high-stringency buffer (RiboWash) at 65°C, the sections were incubated with alkaline phosphatase-conjugated anti-digoxigenin antibody (Roche Diagnostics), and stained with nitro-blue tetrazolium chloride and 5-bromo-4-chloro-3′-indolyl phosphate toluidine salt, and counterstained with fast red.

### Statistical analysis

The significance of the differences between the means was assessed using Student's *t* test except for survival assays. Two-way ANOVA Test was used for analysis of lethality assays.
